# Grief in modern multicultural Europe – a way out of “disenfranchisement”

**DOI:** 10.1192/j.eurpsy.2024.1087

**Published:** 2024-08-27

**Authors:** Z. Correia De Sá, B. Castro Sousa, J. Ramos

**Affiliations:** Department of Psychiatry and Mental Health, Centro Hospitalar Universitário da Cova da Beira, Covilhã, Portugal

## Abstract

**Introduction:**

The recent addition to both ICD-11 and DSM-V of “Prolonged Grief Disorder” “PGD” raises questions regarding the complexity of the clinical manifestations and the nuances of “normal/abnormal” grief. The lack of consensus in diagnosing emphasizes grief as a non-homogeneous process highly dependent on cultural nuances and the proportion of losses.

**Objectives:**

Provide an open discourse on (PGD) emphasizing its multicultural aspects in the diagnosis, and debate whether it reinforces mental health stigma by “pathologizing” grief in today’s multicultural society.

**Methods:**

Non-systematic review of literature using key words *“Grief”, “Prolonged Grief Disorder”, “Multicultural aspects of Grief”, “Major Depressive Disorder” and “Disenfranchised Grief*”, on the platforms PubMed, Medline, Google Scholar, “European Commission”, “International Migration Outlook 2022” and “Pordata”.

**Results:**

Literature has not clearly provided a universal definition of grief, grief processes or the threshold of abnormality. Grief lasting longer than expected is often equated to Major Depressive Episodes, given symptomatic similarities. Migration, war and the pandemic have played a significant role in how people currently grieve. Evidence showed that in 2021 alone there was a 22% increase in the permanent immigrant population. Moreover, the top five nationalities applying for first time asylum in the EU (2022) were: Syrian, Afghan, Venezuelan, Turkish, Columbian, and as of September 2022, 5 million Ukrainian refugees were registered. These figures are not neglectable and show the multiculturality of todays EU population. However, the development of transcultural psychometric tools accessing grief has not been uniform, lacking consistency in validating various transcultural factors. On the other hand, actively diagnosing “Prolonged Grief Disorder” has shown helpful to clinicians in recognizing the debilitating effects of some pernicious grief responses and quickly providing the necessary help.

**Conclusions:**

Diagnosing “PGD” might lead to “psychiatrization” and “medicalization” of normative emotional processes, given different cultural backgrounds, especially due to the absence of universally applicable tools validating transcultural factors. Consequently, an inadequate consideration of context leaves patients feeling invalidated, non-supported and disenfranchised. A culturally sensitive approach is crucial, focusing on individual differences for effective grief intervention and support.

**Disclosure of Interest:**

None Declared

